# Reduced menin expression impairs rapamycin effects as evidenced by an increase in mTORC2 signaling and cell migration

**DOI:** 10.1186/s12964-018-0278-2

**Published:** 2018-10-01

**Authors:** Masoud Razmara, Azita Monazzam, Britt Skogseid

**Affiliations:** Department of medical sciences, Science for Life Laboratory, Uppsala University, Uppsala, Sweden

**Keywords:** MEN1, PNET, PI3K, mTOR, Akt, Rapamycin

## Abstract

**Background:**

Mammalian target of rapamycin (mTOR) is a master regulator of various cellular responses by forming two functional complexes, mTORC1 and mTORC2. mTOR signaling is frequently dysregulated in pancreatic neuroendocrine tumors (PNETs). mTOR inhibitors have been used in attempts to treat these lesions, and prolonged progression free survival has been recorded. If this holds true also for the multiple endocrine neoplasia type 1 (MEN1) associated PNETs is yet unclear. We investigated the relationship between expression of the MEN1 protein menin and mTOR signaling in the presence or absence of the mTOR inhibitor rapamycin.

**Methods:**

In addition to use of menin wild type and menin-null mouse embryonic fibroblasts (MEFs), menin was silenced by siRNA in pancreatic neuroendocrine tumor cell line BON-1. Panels of protein phosphorylation, as activation markers downstream of PI3k-mTOR-Akt pathways, as well as menin expression were evaluated by immunoblotting. The impact of menin expression in the presence and absence of rapamycin was determinate upon Wound healing, migration and proliferation in MEFs and BON1 cells.

**Results:**

PDGF-BB markedly increased phosphorylation of mTORC2 substrate Akt, at serine 473 (S473) and threonine 450 (T450) in menin^−/−^ MEFs but did not alter phosphorylation of mTORC1 substrates ribosomal protein S6 or eIF4B. Acute rapamycin treatment by mTORC1-S6 inhibition caused a greater enhancement of Akt phosphorylation on S473 in menin^−/−^ cells as compared to menin^+/+^ MEFs (116% vs 38%). Chronic rapamycin treatment, which inhibits both mTORC1and 2, reduced Akt phosphorylation of S473 to a lesser extent in menin^−/−^ MEFs than menin^+/+^ MEFs (25% vs 75%). Silencing of menin expression in human PNET cell line (BON1) also enhanced Akt phosphorylation at S473, but not activation of mTORC1. Interestingly, silencing menin in BON1 cells elevated S473 phosphorylation of Akt in both acute and chronic treatments with rapamycin. Finally, we show that the inhibitory effect of rapamycin on serum mediated wound healing and cell migration is impaired in menin^−/−^ MEFs, as well as in menin-silenced BON1 cells.

**Conclusions:**

Menin is involved in regulatory mechanism between the two mTOR complexes, and its reduced expression is accompanied with increased mTORC2-Akt signaling, which consequently impairs anti-migratory effect of rapamycin.

**Electronic supplementary material:**

The online version of this article (10.1186/s12964-018-0278-2) contains supplementary material, which is available to authorized users.

## Background

Pancreatic neroendocrine tumors (PNETs) occur sporadically or inherited as part of the multiple endocrine neoplasia type1 (MEN1) trait caused by inactivating germline mutations in the MEN1 suppressor gene, encoding the protein menin. Gene carriers develop tumors in the parathyroid, the pituitary gland, and in neuroendocrine cells of the pancreas, as well as non-endocrine tumors such as lipomas and facial angiofibromas [1]. Menin is ubiquitously expressed and the organ selectivity of MEN1 associated lesions is unknown. A subset of sporadic PNETs has somatic mutations of the MEN1 gene and others may reveal mutations affecting the mTOR pathway [2]. Most PNETs are malignant and radical surgery is currently the only way to cure the patients. However, advanced disease stages are frequently present already at diagnosis requiring systemic antitumoral therapies. Unfortunately, these therapies show variable but often limited effects [3].

Treatment with mTOR inhibitors has been introduced as an attempt to control malignant progression in patients with PNETs. One comprehensive placebo controlled clinical trial showed prolonged progression free survival in patients with PNETs treated with the orally administered rapamycin analog everolimus (11 vs 4.6 months) [4], but objective tumor responses were few. In our hands, at a tertiary referral center, everolimus is mostly used as second or third line choice in patients with sporadic progressive PNETs. Although stable disease for a limited time is frequently seen, about one third of patients display immediate tumor growth. Adverse events restricting the treatment are also common. Conclusive evidence for using the drug in MEN1 PNET patients has not been presented. Our experience with everolimus in such cases is limited to six patients; adverse reactions and tumor progression within a few months led to drug withdrawal in all but one patient. Although everolimus may successfully be used to control hypoglycemia in insulinoma patients, our experience is that the potential antitumoral effect and the risk of clinically significant side effects are highly unpredictable in the individual patients.

The role of menin in mTOR signaling has not yet been satisfactory established. mTOR is a serine/threonine protein kinase involved in the regulation of different cellular functions, such as cell proliferation, migration, activation of transcription factors and initiation of translation [5]. Growth factors and amino acids can activate the mTOR pathway. mTOR is functional in two distinct multiprotein complexes, namely mTORC1 and mTORC2, each characterized with differential binding partners and substrate specificity. mTORC1 is known as a rapamycin-sensitive complex, containing regulatory-associated protein of mTOR (Raptor). Functions of mTORC1 are to phosphorylate 4EBP1 and activate S6-kinase, which in turn phosphorylates its two downstream targets S6 and eIF4B proteins [6]. Phosphorylated S6 and eIF4B enhance protein syntheses. [7]. mTORC2 consists of rapamycin-insensitive companion of mTOR (Rictor), but details on the mechanism of its activation is yet largely unknown. It has been suggested that mTORC2 activity occurs by PI3-kinase [8]. A negative feedback loop has been described, whereby mTORC1 activation can act as a negative regulator on mTORC2 via insulin or other growth factors and PI3K, for example by reduced PI3K signaling via suppressing insulin receptor substrate-1 (IRS1) [9]. mTORC2 phosphorylates Akt on Ser473 in many cell types [10]. Other substrates for mTORC2 include T450 phosphorylation on Akt, and NDRG1 (N-myc downstream regulated gene 1) [11, 12].

Akt is a serine/threonine kinase which can be activated by serum or growth factors such as platelet derived growth factor (PDGF) in a PI3-kinase-dependent manner [13, 14]. Activated Akt transduces important survival signals that interfere with the apoptotic process, for example inhibition of Foxo and caspase cascade such as caspase 3 and proapoptotic protein BAD [15]. The central importance of Akt signaling appears to be related to hyperactivity of Akt in many type of human cancers [16]. In addition, phosphorylation of Akt is increased in tumors from patients treated with everolimus [17]. It has been shown that PNETs in a MEN1 mice model have increased phosphorylated Akt [18].

Menin is a ubiquitously expressed tumor suppressor protein and its function is only partially understood. It is predominantly localized to the nucleus, but it is also expressed in cell membrane and cytoplasm. In addition to its role in DNA repair and chromatin maintenance, menin is associated with functional nuclear as well as cytoplasmic signals, such as mediating the cytostatic effects of TGFβ and inhibition of transcriptional activation by JunD [19].

In this study, we explore the role of menin in regulation of mTOR signaling pathways, in order to increase knowledge concerning the potential effect of rapamycin analogs in treatment of MEN1-related tumors. The rational for choosing MEF cells for studying the mTOR signaling was that menin^−/−^ MEFs were available [18], MEFs can readily be cultured, and MEN1 patients have fibroblast derived facial angiofibromas. Furthermore, we used a human pancreatic neuroendocrine tumor cell line (BON-1), since rapamycin treatment already is used in patients with PNETs. We confirm that reduction of menin levels promote the activation of mTORC2 signaling. Moreover, in contrast to menin^−/−^ MEFs, we show that extended rapamycin treatment enhances mTORC2-Akt activation when menin is downregulated in BON1 cells. Thus, our data suggest a pivotal key role of menin in mTOR signaling, specifically in transformed pancreatic neuroendocrine cells.

## Methods

### Reagents

Recombinant human PDGF-BB was purchased from Amgen (Thousand Oaks, CA). The inhibitors CI-1040 (PD184352), BAPTA, wortmannin and rapamycin were from Cayman Chemical Company (Michigan, USA). Antibodies against pAkt S473 (#4060), pAkt T450 (#9267), pmTOR (#5536), pS6 (#4858), pErk1/2 (#4370), peIF4B (#3591), pNDRG1 (#3217), pRictorT1135 (#3806), pBad (#9295), Akt (#9272), mTOR (#2972), S6 (#2217). Erk1/2 (#9102), Rictor (#9476), Cleaved caspase 3(#9661) and β-actin (#4970) were purchased from Cell Signaling Technology (Beverly, MA). Menin antibody was purchased from Bethyl Laboratories (Montgomery, USA).

### Cell lines and cell culture

The mouse embryonic fibroblast cell line MEFs were kindly provided by Dr. Sunita K Agarwal (The National Institute of Diabetes and Digestive and Kidney Diseases, Bethesda, Maryland). BON1 cell line, derived from a lymph node metastasis of a PNET producing serotonin, neurotensin, and chromogranin A, was a kind gift from Dr. J.C. Thompson at the Dept of Surgery, University of Texas Medical Branch, Galveston, USA [20]. MEFs and BON1 cells were cultured in Dulbecco’s modified Eagle’s medium (DMEM) and DMEM/F12 respectively, with 10% bovine serum, 100 U/ml penicillin and 100 μg/ml streptomycin. For serum-starvation, cells were washed once and incubated in medium containing 0.1% FBS.

### Immunoblotting and in vivo protein interactions

Subconfluent cells were starved and incubated with vehicle or inhibitors at the indicated concentrations and thereafter stimulated with PDGF-BB (20 ng/ml) or 10% serum for the indicated periods of time. Cells were washed two times in ice-cold phosphate-buffered saline and lysed in RIPA buffer (Sigma) in the presence of protease inhibitor (Cell Signaling Technology (CST), USA). Extracts were clarified by centrifugation, and protein concentration was determined by the BCA protein assay (Pierce). Equal amounts of lysates were boiled with SDS sample buffer containing dithiothreitol. Proteins were separated by SDS-PAGE and then electro-transferred to polyvinylidene difluoride membranes (CST, USA), which were blocked in 5% bovine serum albumin or 5% milk in Tris-buffered saline solution containing 0.1% Tween-20. Primary antibodies were diluted according to the manufacturer’s instructions and membranes incubated overnight at 4 °C. After washing, the membranes were incubated with horseradish peroxidase-conjugated anti-rabbit or anti-mouse IgG antibodies (both from Amersham Biosciences), and proteins were visualized using ECL Plus Western Blotting Detection Systems (GE Healthcare, UK) on a cooled charge-coupled device (CCD) camera (Bio-Rad laboratories, Inc). Densitometrical analysis of the immunoblots was performed and quantified using the Imagelab software (Bio-Rad laboratories, Inc). For immunoprecipitation analysis equal amounts of proteins were incubated overnight at 4 °C with the indicated specific antibodies and corresponding anti-rabbit IgG as negative control, and then incubated with protein G beads for 1 h at 4 °C. The beads were washed 3 times with 1 ml ice cold lysis buffer and 1 time with PBS. The immunocomplexes were eluted from the beads by adding 2× Laemmli SDS-sample buffer upon boiling for 5 min, and then subjected to immunoblot analysis as described above using primary antibodies as specified in the figure legends.

### siRNA knockdown

Downregulation of menin was performed by using 20 nM of specific siRNA (menin ON-TARGETplus SMARTpool) purchased from Dharmacon. For every experiment performed, siRNA (RNA sequence AUGAACGUGAAUUGCUCAA) was used as a control. Transfection of siRNA was done for 48 h with SilentFect from BioRad. Levels of knockdown were tested after 48 h by measuring protein levels by immunoblotting.

### Wound healing assays

Wound healing assays were performed in serum cultured confluent cells growing on non-coated sterile 6-well plates. The cells were treated with or without different inhibitors. A 1000-μl pipette tip was used for scratching, creating an approximately 0.6 mm wide wound. Photomicrographs were obtained by microscope (Axioplan 2; Carl Zeiss MicroImaging, Inc) equipped with a digital camera (C4742–95; Yamaha), using a Plan-neofluar 40 × 0.75 objective lens (Carl Zeiss MicroImaging, Inc). All photographs were taken at room temperature. Primary images were acquired with the camera’s QED software, and analyzed in Tscratch program.

### Transwell migration assay

Twelve-well transwell plates with inserts containing 8-μm pores in a polycarbonate membrane (Sarstedt) were used for the chemotaxis assays. The outer wells contained 1200 μl DMEM containing 10% FBS, as a chemoattractant. Approximately 200 × 10^3^ overnight-starved (1% FBS), either control or menin-siRNA treated BON1 cells in 200 μl FBS-free DMEM, were added to each insert. The transwell plate was then incubated 16 h in an incubator with 5% CO2 at 37 °C. Then the inserts were washed two times with PBS (1X), and cells in the inserts upper surface were gently washed away with PBS (1X) and cleared by ear sticks, and those viable migrated cells trapped under surface of the polycarbonate membrane were trypsinized, and lysed (provided by cell counter supplier) and counted for quantification with an automatic cell counter (NucleoCounter, NC-100; Chemometech).

### Cell number quantification

To determine the number of viable cells after the end of each incubation period, and exposure to the different drugs, the Nucleo Counter NC-100 instrument (ChemoMetec A/S, Denmark) was used. This technology by using compatible Nucleo Cassette is based on detection of fluorescence from DNA-bound fluorescent dye, propidium iodide. Untreated cells were used as control.

### Statistical analysis

All results presented were obtained in at least three independent experiments. Error bars represent the standard error of the mean of triplicate determinations. *P*-values were calculated by using Student’s t-test or two-way ANOVA test with GraphPad Prism, GraphPad Software (Inc., La Jolla, CA, USA), and P-values less than 0.05 were considered statistically significant.

## Results

### Lack of menin enhances mTORC2-Akt, but not mTORC1-S6 activation

**Fig. 1:**
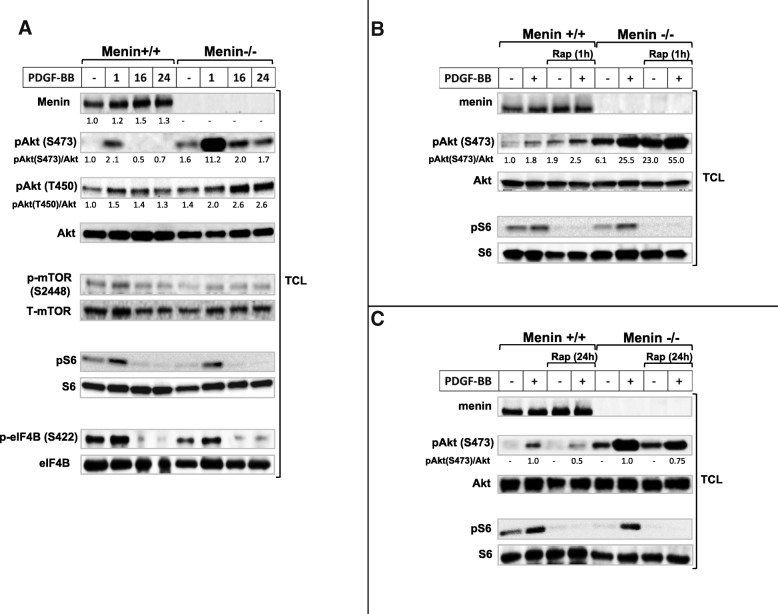
Menin−/− MEFs show enhanced mTORC2, but not mTORC1 activation. Serum-starved menin+/+ and menin−/− MEF cells were cultured in the presence or absence of PDGF-BB (20 ng/ml) at 37 °C for indicated time periods (**a**), or for 30 min (**a** & **b**), with or without pretreatment with the mTOR inhibitor rapamycin (100 nM) for 1 h (**b**) or 24 h (**c**). Total cell lysate (TCL) were analyzed by immunoblotting with antibodies specific for menin, Akt phosphorylated on S473 or T450, or total Akt, as well as mTOR, eIF4B and S6 phosphorylation, and the expression of respective total protein. The relative protein phosphorylations were quantified for a representative experiment. Note enhancement of p-Akt levels, but not p-S6, in menin−/− cells (**a**). Menin−/− cells show enhanced p-Akt after short (1 h) rapamycin treatment (**b**). The inhibitory effect of prolonged (24 h) rapamycin treatment on p-Akt is menin dependent (**c**)

Previous studies on MEFs and many other cell types have shown that mTORC2 is an essential regulator required for activation of Akt by phosphorylation at S473 [10, 21, 22], whereas mTORC1 is responsible for phosphorylation and activation of ribosomal protein S6 [23]. Therefore, we initially employed the use of menin-deficient MEFs as a valuable model in order to determine the molecular effect of menin deletion on mTOR downstream substrates. We found that Akt phosphorylation at S473 is upregulated at the basal level in menin^−/−^ compared to menin^+/+^ MEFs, and PDGF-BB stimulation markedly enhanced Akt phosphorylation at S473 in menin^−/−^, compared to menin^+/+^ MEFs (Fig. 1a).

Additionally, in menin^+/+^ MEFs, at 16 and 24 h with PDGF-BB treatment, Akt phosphorylation of S473 was absent, whiles in menin^−/−^ cells it was still phosphorylated, but returned to basal phosphorylation level (Fig. 1a). Moreover, T450 which recently has been suggested as another target residue of Akt phosphorylation via mTORC2 [9] remains activated at these time points (16 and 24 h), and its activation is more pronounced in the absence of menin (Fig. 1a). These results indicate differential kinetics, duration and residue activation of Akt in menin^−/−^ vs menin^+/+^ MEFs, suggesting elevated Akt activity in the menin^−/−^ cells.

However, mTOR phosphorylation at S2448 has been proposed as a direct target residue of p70S6K [24]. Indeed, we could not observe increased mTOR phosphorylation at S2448 in menin^−/−^ MEFs (Fig. 1a); confirming that S2448 phosphorylation of mTOR occurs independently of the Akt activation.

Furthermore, no upregulation of mTORC1 effectors S6 or eIF4B phosphorylation at the basal, or with PDGF-BB treatment, could be observed in menin^−/−^ compared to menin^+/+^ MEFs (Fig. 1a). This result is in line with previous data that showed S6 phosphorylation occurs independently of mTORC2-Akt activity [21, 25], but in contrast to earlier suggestion of S422 phosphorylation of eIF4B being a direct target of Akt activation [26]. However, the levels of menin expression downstream of PDGF-BB stimulation were slightly enhanced (Fig. 1a), confirming earlier studies that growth factors such as insulin and TGFβ also regulate menin expression [27, 28].

Taken together, menin^−/−^ compared to menin^+/+^ MEFs showed increased mTORC2-Akt activation at the basal level, which was markedly enhanced upon PDGF-BB stimuli. Conversely, absence of menin did not affect the mTORC1-S6 or eIF4B activation.

Moreover, Akt activation by rapamycin has been explained to be through upstream signals in a PI3K-dependet manner [9, 29, 30]. Indeed, PI3K inhibitor wortmannin, and Ca^2+^ chelators BAPTA or EDTA blocked Akt phosphorylation and its enhancement in menin^−/−^ MEFs, but not MAPK inhibitor CI-1040 (Additional file 1: Figure S1A, B and C).

### Lack of menin leads to further enhancement of Akt phosphorylation with rapamycin

**Fig. 2:**
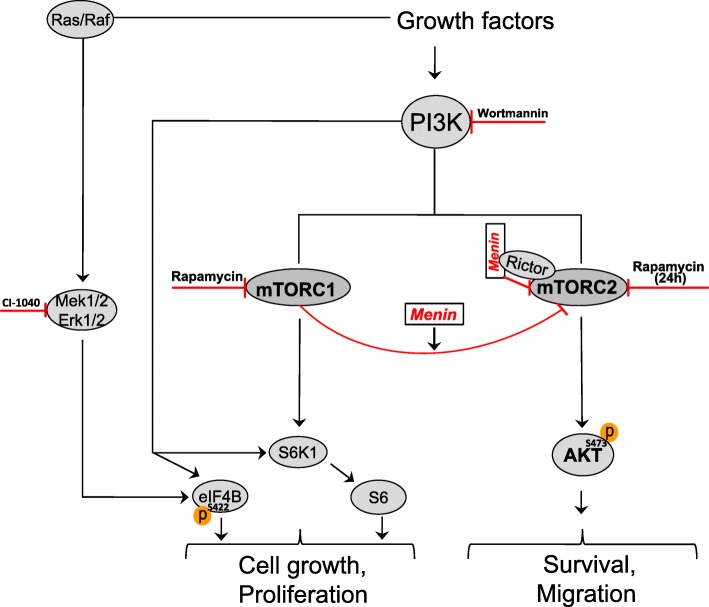
Schematic figure depicting the key roles of menin in mTOR signaling. Initially, PDGF-BB or serum mediated activation of Akt involves PI3K-mTORC2 pathway with menin as negative regulator. Activation of S6 and eIF4B occurs in a PI3K/MAPK dependent manner, but independent of mTORC2-Akt signaling, or menin protein expression. There is a direct negative regulatory interplay between menin protein expression and the rapamycin-mediated activation of mTORC2-Akt

mTOR inhibitors have been used in clinical treatments of several human cancers. However, mTORC1 can act as a negative regulator on mTORC2, since mTORC1 inhibition mediates mTORC2 activation as evidence by elevated Akt phosphorylation at S473 [9, 22, 31]. In order to investigate the possible involvement of menin expression in the inhibitory feedback loop from mTORC1, we treated the menin^−/−^ and menin^+/+^ MEFs with a short rapamycin treatment (1 h), in the presence or absence of PDGF stimulation. Consistent with previous observation that short rapamycin treatment can mediate a feedback activation of Akt [22], a short rapamycin treatment enhanced both basal and PDGF-induced levels of Akt phosphorylation at S473 in menin^+/+^ as well as menin^−/−^ MEFs (Fig. 1b). Interestingly, these enhancements were more obvious in menin^−/−^ cells, 4 fold at basal and 9 fold after PDGF stimulation (Fig. 1b). These results indicate that lack of menin can contribute to rapamycin-induced Akt activation, and this possibly occurs through the loss of negative feedback loop from mTORC1 by rapamycin (Fig. 2).

In contrast to acute rapamycin treatment (1 h), prolonged rapamycin treatment (24 h incubation) has shown to block both mTORC1 and mTORC2 [22]. Consistent with previous study that prolonged rapamycin treatment can inhibit Akt by disrupting mTORC2 [22], 24 h rapamycin treatment reduced the PDGF-induced Akt phosphorylation at Ser473 (Fig. 1c), thus confirming the fact that S473 of Akt is phosphorylated in an mTORC2-dependent manner [10]. Interestingly, this inhibition level (50%) of Akt phosphorylation after 24 h rapamycin treatment upon PDGF stimuli, was markedly lower (25%) in the absence of menin (Fig. 1c), and the Akt phosphorylation did not returned to the basal range of untreated menin^+/+^ MEFs by prolonged rapamycin treatment. Suggesting, absence of menin protein impairs the inhibitory effect of chronic rapamycin treatment on Akt phosphorylation.

As expected, protein S6 phosphorylation was efficiently inhibited by rapamycin treatment (Fig. 1b and c), confirming that menin expression does not interfere with the inhibition of mTORC1-S6 by rapamycin.

Furthermore, our data indicates that menin interact directly with Rictor in mTORC2 (Additional file 1: Figure S2A and B). Hence, this data point out the possible requirement of menin-Rictor complex on the suppression of Akt S473 phosphorylation.

### Inhibition of migration by rapamycin is menin dependent

**Fig. 3:**
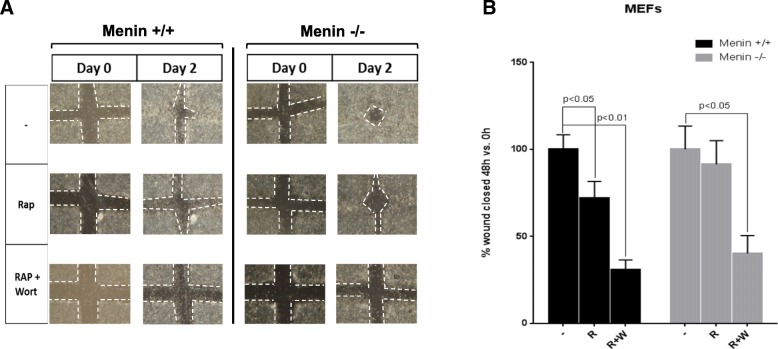
Absence of Menin enhances wound-healing upon rapamycin treatment. Menin−/− and menin+/+ MEFs were subjected to a cell culture wound migration assay as described in Methods. The initial wound space was approximately 0.6 mm. After serum stimulation of cells, or in the presence of rapamycin or dual combination with wortmanin, the cell movement into the gap was imaged with a digital camera using a Zeiss microscope (**a**). The width of the open wound was measured with the T scratcher software. The data are presented as calculated percentage of the wound closure after 48 h relative to corresponding control (**b**). Only in menin+/+ MEFs rapamycin treatment significantly prevented wound healing and cell migration (*P* < 0.05). Data are presented as mean values (± SEM) in 5 independent experiments. Note that rapamycin significantly decreased wound healing in menin+/+ but not in menin−/− cells

Signaling through mTOR has been shown to be center of an oncogenic pathway involved in growth and migrations [32, 33]. Therefore, we aimed to elucidate the functional cellular consequences of menin deletion in migration and proliferation with mTOR inhibitor rapamycin. In a cell cculture wound-healing experiment we used menin^−/−^ and menin^+/+^ MEFs with or without rapamycin, or combination of rapamycin and PI3K inhibitor wortmannin. We found that in the presence of rapamycin menin^−/−^ MEFs show a faster closure of the wounds, as compared to control menin^+/+^ cells (Fig. 3a), suggesting impaired anti-migratory effect of rapamycin in absence of menin.

However, as expected, the migration in both menin^−/−^ and menin^+/+^ MEFs were completely repressed by a combination of drugs. Quantification of five different experiments of the migratory responses of the menin^−/−^ and menin^+/+^ MEFs, with or without rapamycin, or combination of rapamycin and wortmannin, is presented in Fig. 3b.

Furthermore, treatments with rapamycin or wortmannin or their combination significantly reduced the proliferation rate in MEFs, regardless of menin expression levels (Additional file 1: Figure S3A). Thus, taken together these data suggests that absence of menin does not impact the antiproliferative effect of rapamycin but clearly abrogates the anti-migratory effect of the drug.

### Menin downregulation promotes rapamycin-mediated Akt phosphorylation and migration in BON1

**Fig. 4:**
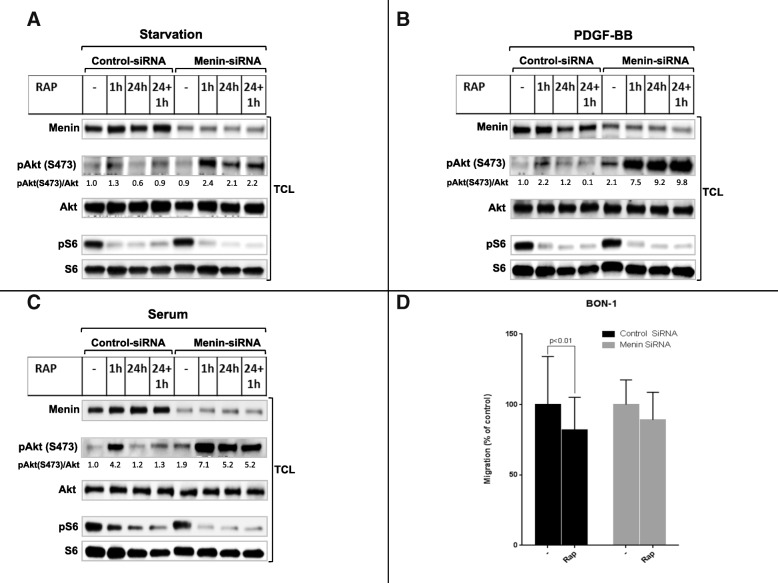
SiRNA-downregulation of menin in BON1 cells enhances Rapamycin-induced Akt phosphorylation and migration. Control or menin-siRNA treated BON1 cells were pretreated with or without rapamycin (100 nM) for indicated time periods upon starvation (**a**), PDGF-BB (**b**), or serum condition (**c**), as specified. Total cell lysate were analyzed by immunoblotting with antibodies specific for menin, Akt phosphorylated on S473, as well as S6 phosphorylation, and the expression of total protein Akt and S6. The relative protein phosphorylations were quantified for a representative experiment. The migration of BON1 cells was carried out as explained in Methods. The 12 well plate were field with medium containing 10% serum as attractant, 200 × 103 pretreated BON1 cells with either control or menin-siRNA, as indicated, were placed on the insert filters to migrate overnight at 37 °C. The amount of the migrated cells is given as percentage relative of corresponding control (**d**). Only in control siRNA treated BON1 cells rapamycin treatment significantly reduced cell migration (*P* < 0.01). Data are presented as mean values (± SEM) in three independent experiments, each performed in triplicate

mTOR signaling is known to be elevated in MEN1 P-NETs [34]. Thus, in order to further confirm the pivotal role of menin in the regulation of mTOR signaling, we used control and siRNA-mediated silencing of menin in the presence and absence of rapamycin in human endocrine pancreatic cells, BON1. Although the downregulation of menin protein expression by siRNA was only partial (50%), we found increased phosphorylation of Akt upon PDGF-BB as well as serum stimulation compared to control siRNA treated BON1 cells (Fig. 4b and c). Thus, menin negatively regulates Akt activation upon PDGF-BB or serum stimulation in BON1 cells.

Consistently, and similar to our results in menin^−/−^ MEFs, 1 h rapamycin treatment specifically elevated Akt activation in BON1 cells when menin levels were reduced by siRNA silencing (Fig. 4a). This enhancement by rapamycin in menin-siRNA treated cells was upregulated with PDGF-BB and serum stimulation (Fig. 4b and c). These results not only reveals the existence of a interwork between the two complexes of mTOR in BON1 cells, but also as in menin^−/−^ MEFs, indicate that menin downregulation contribute to rapamycin-driven Akt activation in BON1 cells.

After siRNA silencing of menin, 24 h rapamycin treatment enhanced the phosphorylation of Akt at S473 in unstimulated BON1 cells (Fig. 4a) and was not able to repress Akt phosphorylation upon serum or PDGF-BB stimulation (Fig. 4b and c), as it did in MEFs (Fig. 1c). Thus, silencing menin correlated with increased short and long-term rapamycin-induced mTORC2-Akt activation in BON1 cells.

To further explore the role of menin in the regulation of mTOR pathway and the signaling interplay between the two mTOR complexes, we also hypothesized that an additional acute rapamycin treatment after a chronic treatment may initiate further enhancement of Akt phosphorylation. Therefore, we treated the BON1 cells after 24 h rapamycin incubation with additional 1 h treatment (24 h + 1 h). Interestingly, similar to 1 h and 24 h rapamycin treatment alone, 24 h + 1 h treatment also enhanced Akt phosphorylation in absence of menin as compared with the control siRNA treated BON1cells (Fig. 4a, b and c).

Collectively, these results might reveal a more pronounced role of menin as suppressor of Akt phosphorylation in rapamycin treated BON1 cells compared to the role of menin in rapamycin treated MEF cells.

As expected, siRNA mediated silencing of menin in BON1 cells did not interfere with the inhibition of mTORC1-S6 activation by rapamycin (Fig. 4a, b and c).

Next, in order to assess migration with another approach, we performed chemotaxis assay in control and menin siRNA treated BON1 cells using serum as chemoattractant in presence or absence of rapamycin. As shown in Fig. 4d, rapamycin treatment significantly (*p* < 0.01) reduced migratory response of control BON1 cells, whereas in BON1 cells with siRNA downregulation of menin the inhibitory effect of rapamycin on migration was impaired (Fig. 4d).

Furthermore, similar to MEFs, treatments with rapamycin or wortmannin or their combination significantly reduced the proliferation rate in BON1 cells (Additional file 1: Figure S3B), regardless of menin expression levels.

These results again confirming that absence of menin may attenuate the antimigratory effect of rapamycin through elevated mTORC2-Akt signaling.

### Readministration of rapamycin retriggers mTORC2 signaling in a menin dependent manner

**Fig. 5:**
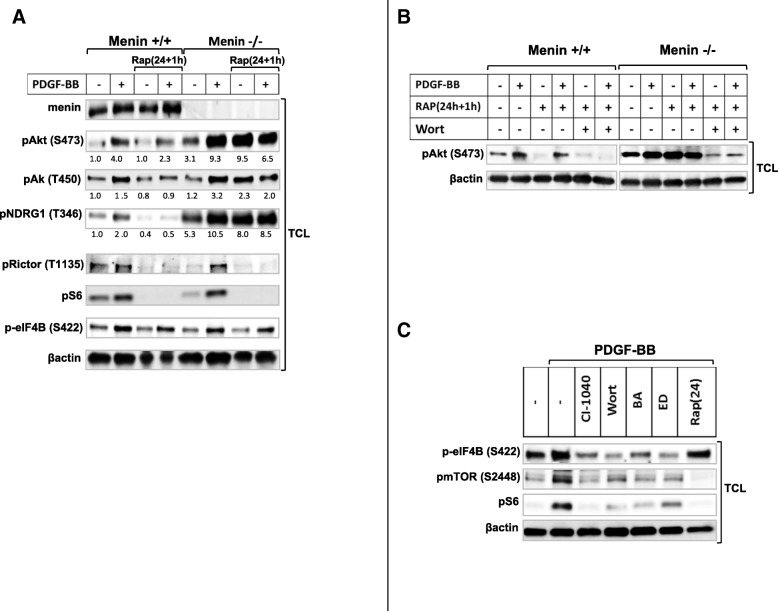
In absence of menin an additional treatment with rapamycin reactivates mTORC2 signaling. Menin+/+ and menin−/− MEFs, were serum-starved for 24 h and then stimulated with PDGF-BB (20 ng/ml) for 30 min or as specified, with or without pretreatment with the mTOR inhibitor rapamycin (100 nM) for 24 h + 1 h (**a**), or in combination with PI3 kinase inhibitor wortmannin (0.2 μm) (**b**). In menin−/− cells rapamycin retreatment markedly increases mTORC2 signaling (**a**). Enhanced rapamycin-induced p-Akt occurs downstream of PI3K (**b**). Menin+/+ MEFs were serum starved and stimulated with PDGF-BB for 30 min after pretreatment with Mek1/2 inhibitor CI-1040, PI3K inhibitor wortmannin (0.2 μm), Ca2+ chelators BAPTA-AM (BA, 10 μM) or EDTA (ED, 2 mM) for 30 min, and rapamycin (100 nM) for 24 h, as indicated (**c**). Total cell lysate were analyzed by immunoblotting with antibodies specific for menin, Akt phosphorylated on S473 and T450, as well as NDRG1, Rictor, S6 and eIF4B phosphorylation, and the expression of βactin. The relative protein phosphorylations were quantified for a representative experiment

Finally, in order to explore our understanding in the involvement of menin expression in the cross talk between the two mTOR complexes we also investigate the effect of the 24 h + 1 h rapamycin treatment in menin^+/+^ and menin^−/−^ MEFs. Results in Fig. 5a and b show, that in the absence of menin and particularly in unstimulated menin^−/−^ MEFs, an additional acute exposure by rapamycin after a prolonged one (24 h + 1 h) was able to retrigger phosphorylation of Akt at S473 (Fig. 5a and b), and other specific targets of mTORC2 signaling such as Akt at T450, and NDRG1 (Fig. 5a). In contrast, 24 h + 1 h rapamycin treatment of menin^+/+^ MEFs potently restrained both basal and PDGF-BB induced mTORC2 signaling (Fig. 5a).

Taken together, these results indicate that menin is required for retaining the inhibitory effect of chronic rapamycin treatment on mTORC2 activation.

Moreover, consistent with lack of menin effect on mTORC1 signaling, we could not observe alterations of T1135 phosphorylation on Rictor in menin^−/-^MEFs (Fig. 5a), whose phosphorylation has been shown to occur downstream of mTORC1 [35]. As expected, rapamycin treatment efficiently reduced PDGF-induced T1135 phosphorylation (Fig. 5a).

PI3K is required for activation of both mTOR complexes [6, 25]. Consistently, S6 and Akt phosphorylation occurred in a PI3K dependent manner, regardless of menin expression (Additional file 1: Figure S1A).

However Akt activity lies downstream of both PI3K and mTORC2, using S473 phosphorylation of Akt as readout for mTORC2 activation, does not really distinguishes PI3K from mTORC2 activity. This rise the question whether absence of menin modulate further enhancement of rapamycin-mediated Akt phosphorylation via PI3K/MAPK, or exert its effect directly on mTORC2. In order to answer this question we used eIF4B, which is a PI3K/MAPK dependet target independent of mTOR. Accordingly a recent study proposes S422 phosphorylation of eIF4B as a rapamycin-resistant residue [36]. Consistently, we could also confirm that phosphorylation of eIF4B occurred PI3K/calcium/MAPK dependently, but independent of mTOR. As shown in Fig. 5c, eIF4B phosphorylation was reduced with either PI3K inhibitor wortmannin or MAPK inhibitor CI-1040, and also with calcium chelators BAPTA or EDTA (Fig. 5c), but it remained still phosphorylated with mTOR inhibitor rapamycin (Fig. 5a and c). Interestingly, an additional short rapamycin exposure after a prolonged one, retriggered mTORC2 signaling only in menin−/− cells, but it did not increase the phosphorylation of eIF4B downstream of PI3K/MAPK (Fig. 5a). This clearly reveals that lack of menin may promote a direct rapamycin mediated mTORC2 signaling independent of PI3K/MAPK. Taken together, our results indicate that menin presumably independent of PI3K is involved in the mTORC1-mediated direct suppression of mTORC2 [31], (Fig. 2).

However, Akt in addition to blockade of caspase 3 cleavage inhibits apoptosis through phosphorylation and inactivation of several targets, including Bad [15]. Absence of menin in MEF cells led to marked Bad phosphorylation (Additional file 1: Figure S4B) and inhibition of cleaved caspase-3 upon mitogen response (Additional file 1: Figure S4A). Importantly, rapamycin was unable to stimulate apoptosis in menin^−/−^ cells as evidence by enhanced phosphorylation of BAD (Additional file 1: Figure S4B).

## Discussion

mTOR controls various cellular mechanisms through mTORC1 and mTORC2 involving series of regulatory feedback loops. It is suggested that phosphorylation at S473 and T450 of Akt occurs via mTORC2 [10], whereas mTORC1 phosphorylates S6 and eIF4B [6, 37, 38]. Here, we report that phosphorylation level of Akt at S473 was critically dependent on menin expression since the phosphorylation of S473 was strongly elevated in menin^−/−^ MEFs and upon mitogen stimulation when menin was downregulated in the human pancreatic endocrine cell line BON1. A recent study showed that overexpression of menin leads to reduced Akt accessibility at the cell membrane to be phosphorylated [18]. However, in menin^−/−^ cells we could also show that other activation markers of mTORC2 such as Akt at T450 and NDRG1 [11, 12] are following the same menin-dependant phosphorylation pattern as S473. This strongly implies an inhibitory role of menin on mTORC2.

Activated mTORC1 elicits a potent negative feedback loop to suppress mTORC2-Akt activation [9, 22, 31], and upon treatment with mTORC1 inhibitors such as rapamycin the loss of this negative signal on mTORC2 will lead to Akt phosphorylation. Several studies have shown that this occurs by upstream signaling via PI3K [9, 29], whiles recent evidence suggests that mTORC1 can directly regulate mTORC2 activation [31, 35]. Here we found that short term-rapamycin treatment enhanced Akt phosphorylation in both endocrine and nonendocrine cells in a menin-dependent manner, suggesting involvement of menin protein in the crosstalk between mTORC1 and 2.

In contrast to short-term, long-term rapamycin treatment in some cell types has been shown to have inhibitory effect on Akt phosphorylation at S473, possibly by interfering directly with mTORC2 [22]. Our results however showed that the effect of extended treatment with rapamycin on mTORC2-Akt activation is not only menin dependent, but also cell type-specific. For instance, in menin^−/−^ MEF cells prolonged rapamycin treatment failed to represses PDGF-induced Akt activation with similar efficiency as it did in menin^+/+^ MEFs. Whereas, in BON1 cells when menin was only partially silenced (50%), rapamycin did not longer block, but triggered further Akt activation. Although, PNETs and angiofibromas are equally common in MEN1 patients, BON1 cells and MEFs show variable response to rapamycin, and BON1 cells with downregulated menin display a more pronounced rapamycin resistance phenotype.

Since treatment with mTOR inhibitors, such as everolimus, has become clinical practice in many centers for management of patients with malignant PNETs this finding of menin dependent rapamycin resistance could be of immense clinical importance. However, the possibility to further examine the functional significance of our findings on proliferation and tumorigenicity of menin dependent rapamycin effects in a PNET cell line is seriously hampered by the lack of available menin-deleted human PNET cell line. Our findings do however warrant caution when using mTOR inhibitors for treatment of MEN1 associated PNETs, and the effect needs to be thoroughly investigated in comprehensive prospective clinical studies.

We provide data indicating that menin interact directly with Rictor. Furthermore, it has been suggested that Rictor in mTORC2 is assembled in such a way that the rapamycin binding site of mTOR is not accessible to the drug, thus rapamycin cannot bind to an already preformed rictor-mTOR complex [22]. Notably, microarray data from hz MEN1 mice islets compare to wt littermates mice, showed upregulation of Rictor (but not Raptor) [39]. However, during long-term rapamycin treatment the drug is able to bind to newly synthesized mTOR, before mTOR assembles into mTORC2 [22]. Hence, one could speculate that menin-Rictor interaction prevents Rictor to occupy rapamycin binding site of mTOR, thereby contribute to inhibitory effect of prolonged rapamycin treatment on mTORC2-Akt activity. This could in part explain why the inhibitory effect of rapamycin on Akt activation is hampered by lack of menin expression. However, the specific role of menin-Rictor interaction remains to be elucidated.

mTORC1 and mTORC2 can function independently to maintain cellular events. Targeting mTORC2 prevents cell migration and promotes apoptosis [40] whiles blocking mTORC1 signaling has been reported to regulate proliferation [31]. Thus, biological effects of the menin-dependent rapamycin effects on mTOR signaling should be possible to assess by analyzing cell growth and proliferation (mTORC1 activity), as well as survival/apoptosis and migration (mTORC2 activity). Accordingly, in our hands extended rapamycin treatment of fibroblasts (MEFs), and pancreatic endocrine tumor cells (BON1), potently reduced cell migration only in the presence of menin, whereas low levels or complete lack of menin decreased the effects of rapamycin. Therefore, menin by modulating mTORC2 signaling seems to be critical for rapamycin effect on migration. On the other hand, since our results show that menin is not involved in mTORC1 inhibition by rapamycin, we were not surprised to find that growth inhibition in response to rapamycin was independent of menin expression. However, involvement of other signal mechanism(s) not identified in this study can be considered.

## Conclusions

This study suggests that menin is essential in the crosstalk between mTORC1 and mTORC2, as evidenced by its requirement for maximal rapamycin effect on mTORC2 signaling and cell migration (Fig. 2). The latter might influence the use of mTOR inhibitors in management of patients suffering from pancreatic endocrine tumors with complete lack of MEN1 gene expression.

## Additional file

Additional file 1: **Figure S1.** Enhanced Akt phosphorylation in absence of menin is PI3K-Ca^+ 2^ dependent, but MAPK independent. **Figure S2.** Rictor in mTORC2 interacts with menin. **Figure S3.** Absence of menin or its downregulation do not affect the proliferation upon rapamycin treatment. **Figure S4.** Absence of menin reduces apoptotic signals. (PPTX 448 kb)

## Abbreviations

Erk: Extracellular regulated kinase
MAPK: Mitogen activated protein kinase
MEF: Mouse embryo fibroblast
MEN1: Multiple endocrine neoplasia type 1
mTOR: Mammalian target of rapamycin
PDGF: Platelet-derived growth factor
PI3K: Phosphatidylinositol 3-kinase
PNETs: Pancreatic neuroendocrine tumors
