# Supplementary material for: Reduced menin expression impairs rapamycin effects as evidenced by an increase in mTORC2 signaling and cell migration
Source: Cell Commun Signal. 2018 Oct 1;16:64. doi: 10.1186/s12964-018-0278-2 (PMC6167842; doi:10.1186/s12964-018-0278-2)
Supplement: Supplementary file 1 — Figure S1. Enhanced Akt phosphorylation in absence of menin is PI3K-Ca+ 2 dependent, but MAPK independent. Figure S2. Rictor in mTORC2 interacts with menin. Figure S3. Absence of menin or its downregulation do not affect the proliferation upon rapamycin treatment. Figure S4. Absence of menin reduces apoptotic signals. (PPTX 448 kb) [file 12964_2018_278_MOESM1_ESM.pptx]

## Slide 1
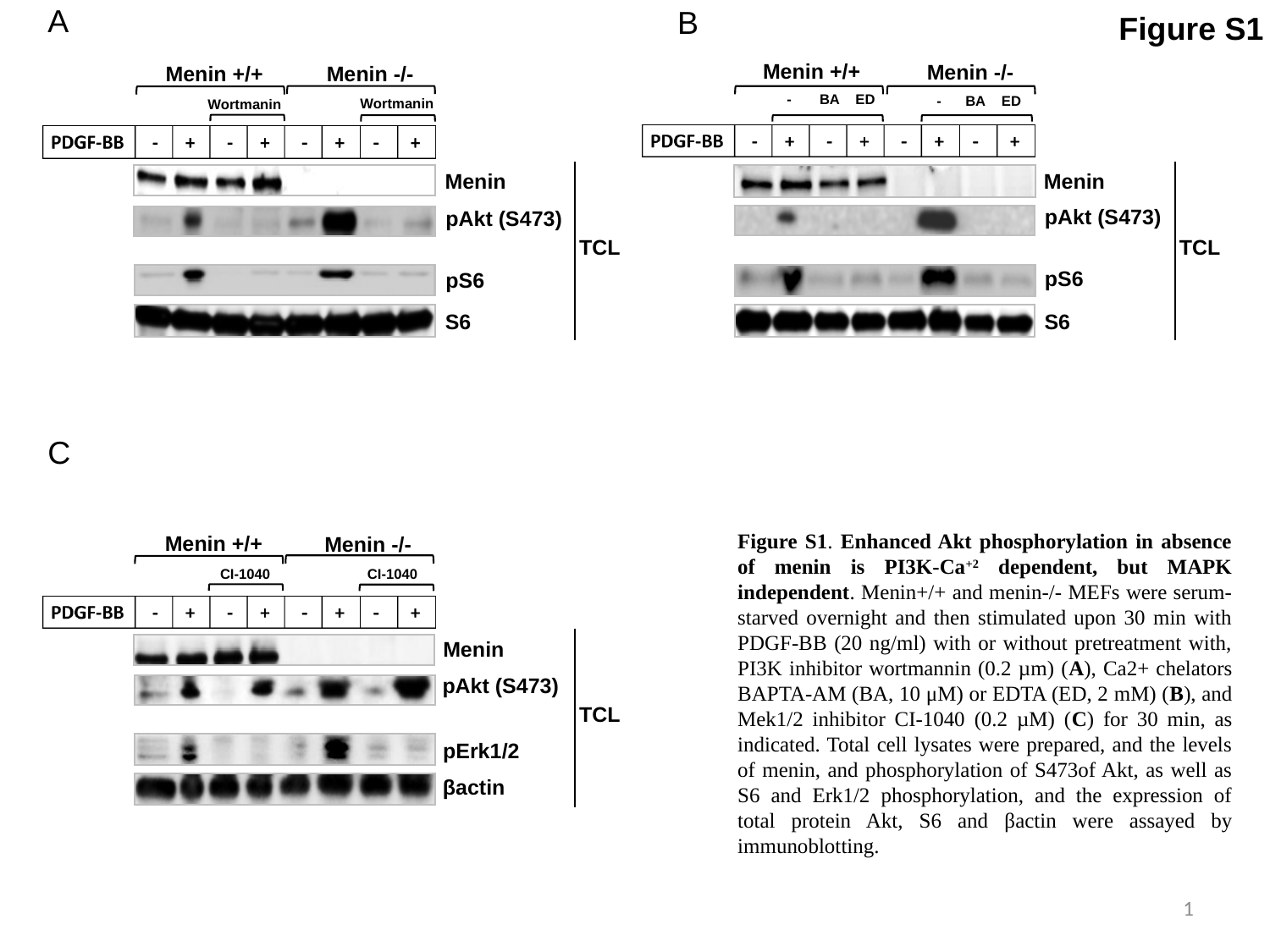

A
B
Figure S1
Menin +/+
Menin -/-
Menin +/+
Menin -/-
 - BA ED
- BA ED
Wortmanin
Wortmanin
Menin
Menin
pAkt (S473)
pAkt (S473)
TCL
TCL
pS6
pS6
S6
S6
C
Figure S1. Enhanced Akt phosphorylation in absence of menin is PI3K-Ca+2 dependent, but MAPK independent. Menin+/+ and menin-/- MEFs were serum-starved overnight and then stimulated upon 30 min with PDGF-BB (20 ng/ml) with or without pretreatment with, PI3K inhibitor wortmannin (0.2 µm) (A), Ca2+ chelators BAPTA-AM (BA, 10 μM) or EDTA (ED, 2 mM) (B), and Mek1/2 inhibitor CI-1040 (0.2 µM) (C) for 30 min, as indicated. Total cell lysates were prepared, and the levels of menin, and phosphorylation of S473of Akt, as well as S6 and Erk1/2 phosphorylation, and the expression of total protein Akt, S6 and βactin were assayed by immunoblotting.
Menin +/+
Menin -/-
CI-1040
CI-1040
Menin
pAkt (S473)
TCL
pErk1/2
βactin
1

## Slide 2
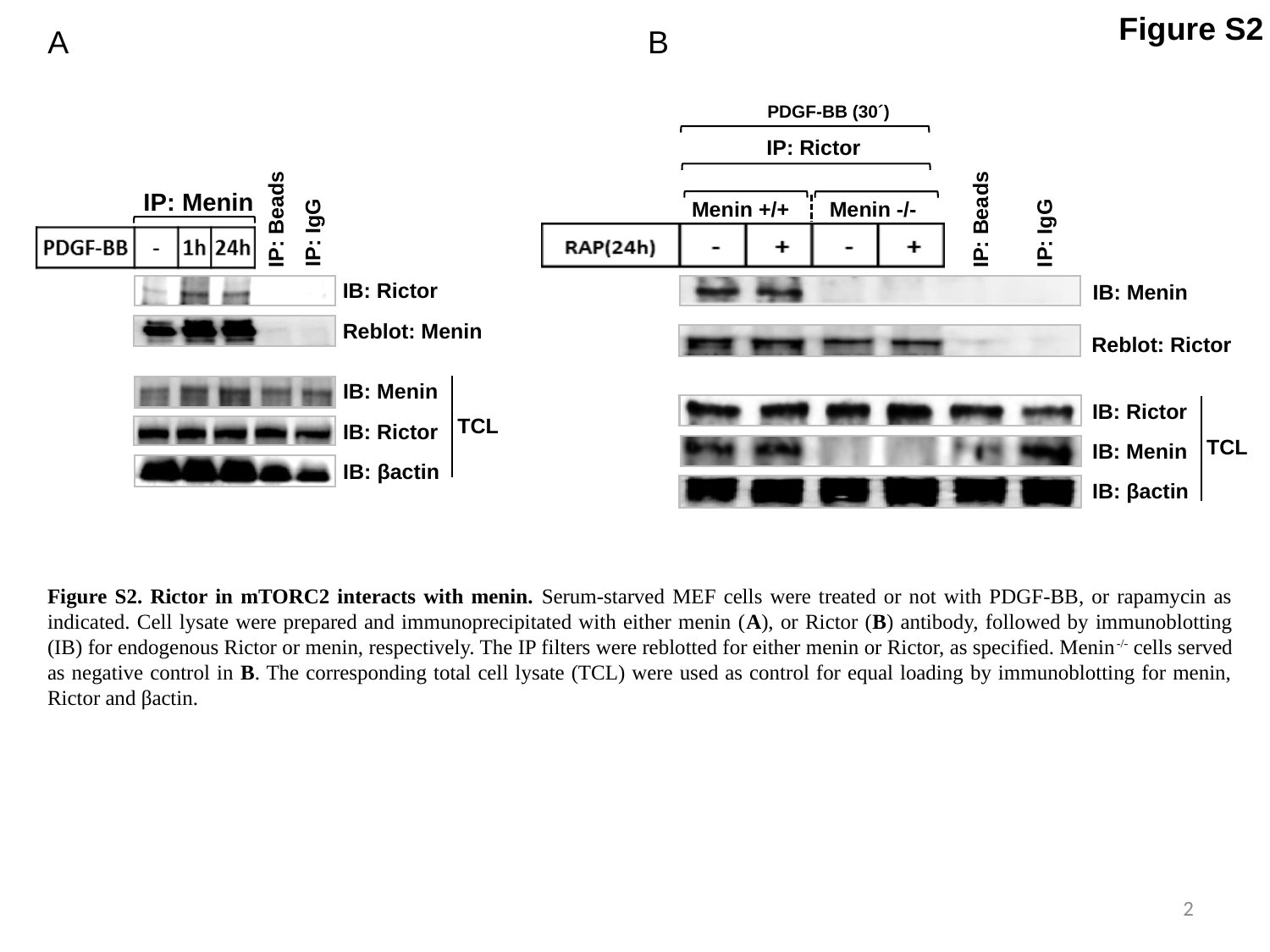

Figure S2
A
B
PDGF-BB (30´)
IP: Rictor
IP: Menin
Menin +/+
Menin -/-
IP: IgG
IP: Beads
IP: Beads
IP: IgG
IB: Rictor
IB: Menin
Reblot: Menin
Reblot: Rictor
IB: Menin
IB: Rictor
TCL
IB: Rictor
TCL
IB: Menin
IB: βactin
IB: βactin
Figure S2. Rictor in mTORC2 interacts with menin. Serum-starved MEF cells were treated or not with PDGF-BB, or rapamycin as indicated. Cell lysate were prepared and immunoprecipitated with either menin (A), or Rictor (B) antibody, followed by immunoblotting (IB) for endogenous Rictor or menin, respectively. The IP filters were reblotted for either menin or Rictor, as specified. Menin-/- cells served as negative control in B. The corresponding total cell lysate (TCL) were used as control for equal loading by immunoblotting for menin, Rictor and βactin.
2

## Slide 3
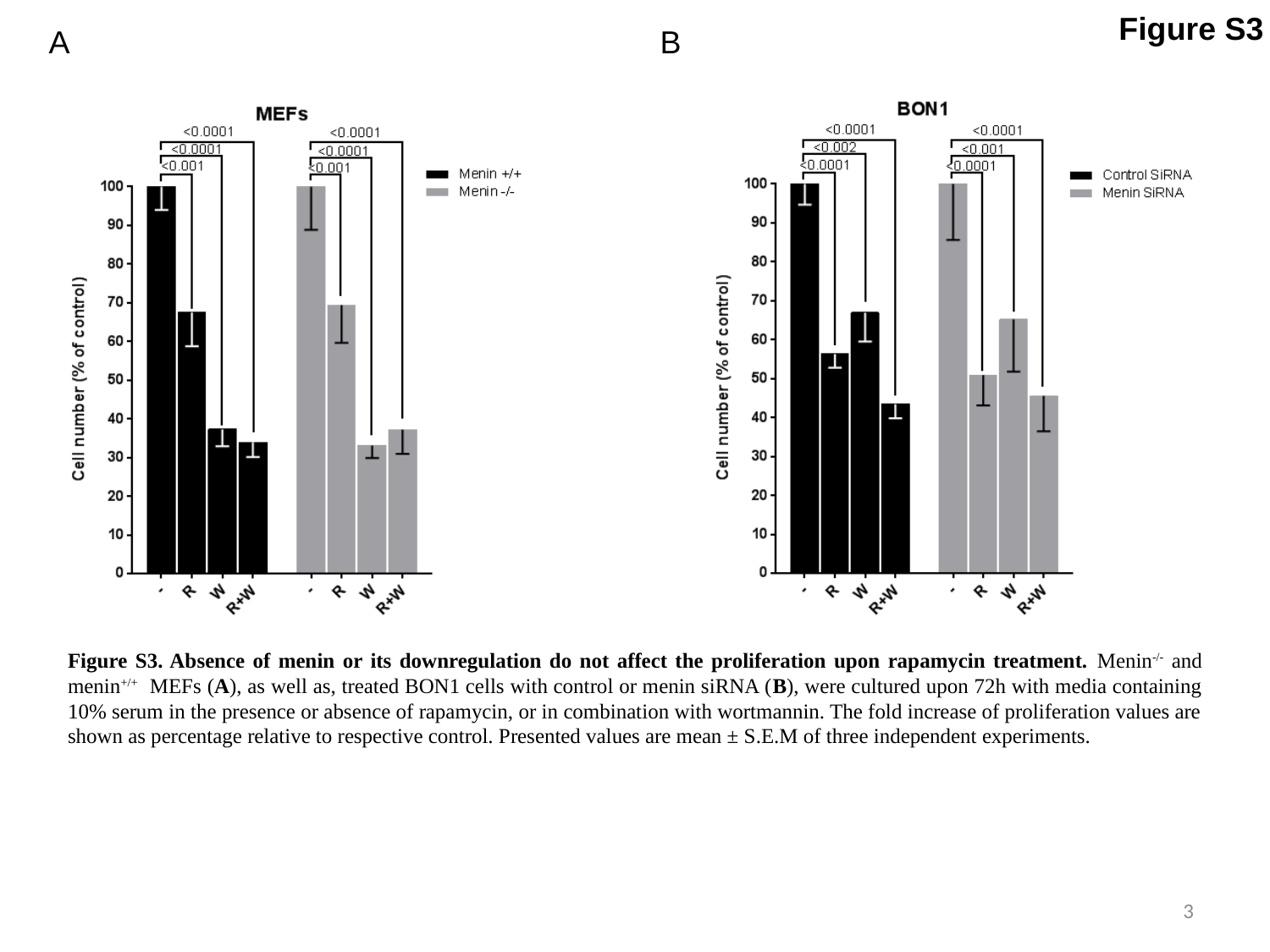

Figure S3
A
B
Figure S3. Absence of menin or its downregulation do not affect the proliferation upon rapamycin treatment. Menin-/- and menin+/+ MEFs (A), as well as, treated BON1 cells with control or menin siRNA (B), were cultured upon 72h with media containing 10% serum in the presence or absence of rapamycin, or in combination with wortmannin. The fold increase of proliferation values are shown as percentage relative to respective control. Presented values are mean ± S.E.M of three independent experiments.
3

## Slide 4
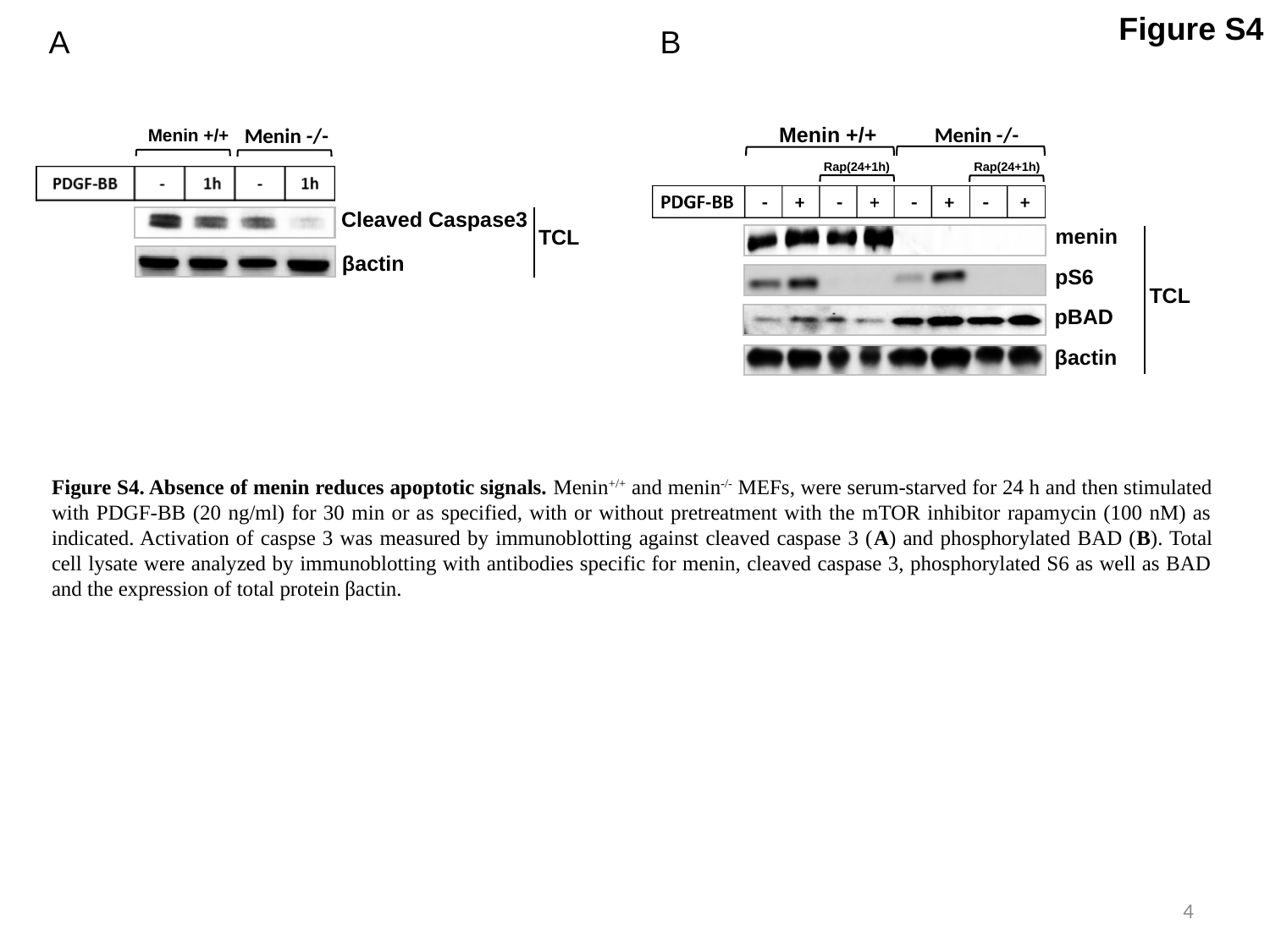

Figure S4
A
B
Menin +/+
Menin -/-
Menin -/-
Menin +/+
 Rap(24+1h)
Rap(24+1h)
 Cleaved Caspase3
menin
TCL
βactin
pS6
TCL
pBAD
βactin
Figure S4. Absence of menin reduces apoptotic signals. Menin+/+ and menin-/- MEFs, were serum-starved for 24 h and then stimulated with PDGF-BB (20 ng/ml) for 30 min or as specified, with or without pretreatment with the mTOR inhibitor rapamycin (100 nM) as indicated. Activation of caspse 3 was measured by immunoblotting against cleaved caspase 3 (A) and phosphorylated BAD (B). Total cell lysate were analyzed by immunoblotting with antibodies specific for menin, cleaved caspase 3, phosphorylated S6 as well as BAD and the expression of total protein βactin.
4
